# Human and Animal Trypanosomes in Côte d'Ivoire Form a Single Breeding Population

**DOI:** 10.1371/journal.pone.0067852

**Published:** 2013-07-02

**Authors:** Paul Capewell, Anneli Cooper, Craig W. Duffy, Andy Tait, C. Michael R. Turner, Wendy Gibson, Dieter Mehlitz, Annette MacLeod

**Affiliations:** 1 Wellcome Trust Centre for Molecular Parasitology, College of Medical, Veterinary and Biological Sciences, Glasgow, United Kingdom; 2 College of Medical, Veterinary and Biological Sciences, Glasgow, United Kingdom; 3 School of Biological Sciences, University of Bristol, Bristol, United Kingdom; 4 Institute for Parasitology and Tropical Veterinary Medicine, Freie Universität Berlin, Berlin, Germany; Institut national de la santé et de la recherche médicale - Institut Cochin, France

## Abstract

**Background:**

*Trypanosoma brucei* is the causative agent of African Sleeping Sickness in humans and contributes to the related veterinary disease, Nagana. *T. brucei* is segregated into three subspecies based on host specificity, geography and pathology. *T. b. brucei* is limited to animals (excluding some primates) throughout sub-Saharan Africa and is non-infective to humans due to trypanolytic factors found in human serum. *T. b. gambiense* and *T. b. rhodesiense* are human infective sub-species. *T. b. gambiense* is the more prevalent human, causing over 97% of human cases. Study of *T. b. gambiense* is complicated in that there are two distinct groups delineated by genetics and phenotype. The relationships between the two groups and local *T. b. brucei* are unclear and may have a bearing on the evolution of the human infectivity traits.

**Methodology/Principal Findings:**

A collection of sympatric *T. brucei* isolates from Côte d’Ivoire, consisting of *T. b. brucei* and both groups of *T. b. gambiense* have previously been categorized by isoenzymes, RFLPs and Blood Incubation Infectivity Tests. These samples were further characterized using the group 1 specific marker, *TgSGP*, and seven microsatellites. The relationships between the *T. b. brucei* and *T. b. gambiense* isolates were determined using principal components analysis, neighbor-joining phylogenetics, STRUCTURE, F_ST_, Hardy-Weinberg equilibrium and linkage disequilibrium.

**Conclusions/Significance:**

Group 1 *T. b. gambiense* form a clonal genetic group, distinct from group 2 and *T. b. brucei*, whereas group 2 *T. b. gambiense* are genetically indistinguishable from local *T. b. brucei*. There is strong evidence for mating within and between group *2 T. b. gambiense* and *T. b. brucei*. We found no evidence to support the hypothesis that group 2 *T. b. gambiense* are hybrids of group 1 and *T. b. brucei*, suggesting that human infectivity has evolved independently in groups 1 and 2 *T. b. gambiense*.

## Introduction

The parasite *Trypanosoma brucei* is the causative agent of African Sleeping Sickness in humans and one of several pathogens that cause the veterinary disease Nagana. These diseases have a wide distribution across sub-Saharan Africa and affect some of the poorest areas of the world. *T. brucei* is traditionally segregated into three morphologically identical sub-species based on host, geography and pathology. *T. b. brucei* is limited to domestic and wild animals throughout sub-Saharan Africa and is non-infective to humans (and some primates) due to sensitivity to trypanosome lytic factors found in their serum [1]. *T. b. gambiense* and *T. b. rhodesiense* are human infective sub-species, named due to their relative geographic locations. While *T. b. rhodesiense* can infect humans due to possession of a serum resistance associated (*SRA*) gene [2], this gene is not present in the more prevalent West and central African trypanosome subspecies, *T. b. gambiense* [3]–[5], causative agent of over 95% of reported cases of sleeping sickness [6], [7]. How this sub-species is able to resist lysis by human trypanolytic factors is still unknown, although there is a decrease in uptake of lytic factor due to modification of the HpHbR cell surface receptor that contributes to resistance [8]–[12]. The study of *T. b. gambiense* is complicated in that there are two distinct groups within the sub-species that can be delineated by genetics and several phenotypic characteristics [13]. Group 1 *T. b. gambiense* causes a chronic infection, is invariably resistant to human serum and is by far the more prevalent group of the two. It appears to be largely a disease limited to humans, although some animal reservoirs have been described [14]–[19]. Conversely, group 2 *T. b. gambiense* are reported to be more virulent and exhibit a variable human serum resistance mechanism in a manner similar to *T. b. rhodesiense* [16], [19]. Unlike group 1 *T. b. gambiense*, group 2 *T. b. gambiense* does not possess the modification to the cell surface receptor HpHbR to avoid lysis by human serum and appears to utilize a different method to counter lytic factors that is distinct from group 1 *T. b. gambiense* or *T. b. rhodesiense* [20]. This group of *T. b. gambiense* has only been described in Côte d’Ivoire, Cameroon and Burkina Faso to date, and always in geographical areas where group 1 *T. b. gambiense* is also found [13], [21]. This geographical overlap of the two groups of *T. b. gambiense* raises interesting questions as to whether they share a close genetic relationship and possibly the ability to undergo sexual recombination.

Investigation of the population genetics of *T. b. gambiense* would reveal if such a relationship exists, however most research on *T. b. gambiense* field populations has focused on the more prevalent group 1. Studies with isoenzymes and RFLPs have indicated that group 1 *T. b. gambiense* populations exhibit low genetic variation and appear distinct from *T. b. brucei* populations [16], [19], [21]–[26] and recent comprehensive microsatellite genotyping techniques appear to have confirmed this for some populations [27], [28]. This is in contrast to high genetic variation found in *T. b. brucei* populations [16], [19], [22]–[26], [29]. It has also been shown that while group 1 *T. b. gambiense* are clonal within a disease focus, there are genetic differences between isolates from different geographic locations [27]. During the decades of investigation into the population structure of *T. brucei*, a second group of human infective *T. b. gambiense* from Côte d’Ivoire and Burkina Faso (formally Upper Volta) were identified that possess different isoenzyme and RFLP profiles from the classical group 1 *T. b. gambiense* isolates. These isolates also showed greater genetic variation and appeared more similar to local *T. b. brucei* [16], [19], [22]–[26], [30]. The greater variation in genetic markers and similarity to the *T. b. brucei* population suggested that this second group of *T. b. gambiense* might be genetically competent, in contrast to group 1. This has subsequently been confirmed by laboratory crosses between *T. b. gambiense* group 2 and both *T. b. brucei* and *T. b. rhodesiense* strains [31]–[33]. The possibility that different mating structures may exist in the two groups of *T. b. gambiense* has implications in evaluating the relationships between them and the evolution of several traits in the population, including human infectivity. It is possible that this trait has evolved twice in separate populations of *T. b. gambiense* or conversely evolved once but due to mating events has become invariant in group 1 and variable in group 2 *T. b. gambiense*.

A recent study of microsatellite multi-locus genotypes from many *T. brucei* isolates from across Africa has found that group 1 *T. b. gambiense* form a clade separate from *T. b. brucei*, *T. b. rhodesiense* and group 2 *T. b. gambiense* [29]. Group 2 *T. b gambiense* individuals formed a cluster positioned between *T. b. brucei* and group 1 *T. b. gambiense*, suggesting that group 2 *T. b. gambiense* may be a hybrid of group 1 and *T. b. brucei* and would perhaps share a mechanism for human infectivity. However, the *T. b. brucei* used in the study were mostly from East Africa and are not representative of West African *T. b. brucei* that are sympatric with the two *T. b. gambiense* groups. In order to resolve the relationships between the two groups of *T. b gambiense* and local *T. b. brucei*, we used a microsatellite genotyping approach to examine a collection of *T. brucei* isolates from Côte d’Ivoire [15], [16], [34]. The isolates have been characterized by the Blood Incubation Infectivity Test (BIIT) [35], [36] and defined as highly resistant (all of 5 rodents infected), intermediate or sub-resistant (1–4 rodents were infected) or sensitive (none of 5 rodents infected) (Table S1). They have also been typed as classical gambiense (group 1) or non-gambiense by isoenzyme or RFLP profiles [16], [21]. All of these samples were collected from a similar time period (1978–1983) and from a geographic area that contains both groups of *T. b. gambiense*. This collection of isolates allows the analysis of the sub-species genetics at this time point and the elucidation of the relationships between them, especially those between group 1 and 2 *T. b. gambiense,* which has implications for the evolution of human infectivity traits.

## Materials and Methods

### Ethical Statement

Ethical approval for the human derived isolates in this study have been previously published [16], [17].

### Isolate Library

The collection of 43 *T. brucei* isolates used for population analysis was collected from a disease focus in Côte d’Ivoire that encompasses the townships of Gagnoa, Vavoua, Daloa and Bouaflé [16], [21] from a range of hosts, including humans, between 1978 and 1983 (Table 1) The townships are approximately 50km to 90km apart. Samples were obtained as purified DNA or blood spots from stabilates on FTA cards that were washed and prepared as per the manufacturer’s instructions before analysis (Whatman). The collection comprises both classical group 1 *T. b. gambiense* and group 2 *T. b. gambiense* as identified by isoenzymes and RFLPs [35], [36], and also non-human infective *T. b. brucei* determined by BIIT. A 5 rodent BIIT [37] was used to assess whether each isolate possessed the potential for human infectivity [11]. In addition to the main sample collection, the following reference isolates were also included: the group 1 *T. b. gambiense* genome strain MHOM/CI/86/DAL972 [27], [38], the 2 *T. b gambiense* strain STIB386 (MHOM/CI/78/TH114/78E) (also included in the main collection [39]) used in the *T. b. gambiense* genetic map [27], [38] and the comprehensively studied group 1 *T. b. gambiense* strain MHOM/CI/52/ELIANE were also included.

**Table 1:** Details of the isolates used in the study, including the isolate name, the host each strain was isolated from, the serum resistance profile resulting from a 5 rodent BIIT (R = highly resistant, I = intermediate or sub-resistant and S = sensitive) and the geographic location of the isolate when collected within Côte d’Ivoire.

Isolate Name	Host	Serum Resistance	Location
**MHOM/CI/78/TH112**	Human	S	Bouafle
**MHOM/CI/78/TH114**	Human	R	Bouafle
**MHOM/CI/78/TH126**	Human	I	Bouafle
**MSUS/CI/78/TSW065**	Pig	S	Bouafle
**MSUS/CI/78/TSW100**	Pig	I	Bouafle
**MSUS/CI/78/TSW113**	Pig	I	Bouafle
**MSUS/CI/78/TSW115A**	Pig	R	Bouafle
**MSUS/CI/78/TSW168**	Pig	I	Bouafle
**MSUS/CI/78/TSW175**	Pig	R	Bouafle
**MSUS/CI/78/TSW178**	Pig	S	Bouafle
**MSUS/CI/78/TSW18**	Pig	I	Bouafle
**MSUS/CI/78/TSW182**	Pig	I	Bouafle
**MSUS/CI/78/TSW19**	Pig	I	Bouafle
**MSUS/CI/78/TSW190**	Pig	I	Bouafle
**MSUS/CI/78/TSW196**	Pig	S	Bouafle
**MSUS/CI/78/TSW209**	Pig	I	Bouafle
**MSUS/CI/78/TSW251**	Pig	S	Bouafle
**MSUS/CI/78/TSW308**	Pig	I	Bouafle
**MSUS/CI/78/TSW332**	Pig	S	Bouafle
**MSUS/CI/78/TSW38**	Pig	S	Bouafle
**MSUS/CI/78/TSW390**	Pig	S	Bouafle
**MSUS/CI/78/TSW65**	Pig	S	Bouafle
**MSUS/CI/78/TSW77**	Pig	I	Bouafle
**MHOM/83/DAL587**	Human	R	Bouafle
**MHOM/83/DAL598**	Human	R	Daloa
**MHOM/83/DAL642**	Human	R	Daloa
**MHOM/83/DAL645**	Human	R	Daloa
**MHOM/83/DAL403**	Human	R	Daloa
**MHOM/83/DAL633**	Human	R	Daloa
**MHOM/CI/86/DAL972**	Human	R	Daloa
**MHOM/CI/82/DAL654**	Human	R	Daloa
**MHOM/CI/83/DAL596**	Human	R	Daloa
**MHOM/CI/83/DAL607**	Human	R	Daloa
**MHOM/CI/78/DAL069**	Human	R	Daloa
**MHOM/CI/78/DAL072A**	Human	R	Daloa
**MHOM/83/DAL595**	Human	R	Gagnoa
**MHOM/CI/83/DAL543**	Human	R	Vavoua
**MHOM/CI/78/TH1**	Human	S	Vavoua
**MSUS/CI/78/TSW155**	Pig	S	Vavoua
**MSUS/CI/78/TSW158**	Pig	I	Vavoua
**MSUS/CI/78/TSW187**	Pig	I	Vavoua
**MHOM/83/DAL542**	Human	R	Vavoua
**MHOM/84/DAL740**	Human	R	Vavoua
**MHOM/CI/82/DAL494**	Human	R	Vavoua
**MHOM/CI/52/ELIANE**	Human	R	Côte d’Ivoire

### Genotyping

Samples were genotyped for population analysis using previously published nested PCR primers for eight microsatellite markers; Ch1/18, Ch1/D2/7, Ch2/PLC, Ch2/5, Ch5/JS2, Ch11/110, Ch3/IJ15/1 and Ch4/M12C12 [40]. The Ch4/M12C12 marker proved to be monomorphic for these samples and was excluded. Markers on the same chromosome are at opposite ends of the chromosome to each other and are unlikely to be linked [27]. All of the primers used to amplify the microsatellites have been previously identified and verified for both *T. b. brucei* and *T. b. gambiense* populations [41]. The PCR conditions for each nested reaction were 95°C 50 secs, 55°C 50 secs and 65°C 60 secs, for 30 cycles. PCR was performed in a total volume of 30 µl with the primers at a final concentration of 10 µM each and Taq polymerase final concentration of 0.25 units/µl (Thermo Scientific). If a sample was homozygous at a marker, it was repeated, otherwise all reactions were performed once. To determine allele size, one of the second round primers was tagged using either AFAM or HEX fluorescent dye to allow accurate sizing on a capillary ABI sequencer against ROX labeled size standards (Dundee Sequencing Unit). The size of the tagged PCR product was measured to within 2bp using the Peak Scanner® software package (Applied Biosystems). Each distinct allele size was given a number and the two alleles for each locus were catalogued for each isolate. This allowed the creation of a multi-locus genotype (MLG) for each isolate. In addition to the microsatellite MLG, the samples were further characterized using the previously published diagnostic PCR for the presence/absence of the *TgSGP* gene [42], [43] and an improved set of nested *TgSGP* primers that span the 5′ and 3′ regions of the gene [44]. The PCR conditions used for these primers were 95°C 50 secs, 55°C 50 secs and 65°C 120 secs, for 35 cycles. *TgSGP* PCR was performed in a total volume of 10 µl.

### Population Analysis

The multi-locus genotype (MLG) of each isolate studied was used to create a dendrogram to infer relationships within the Côte d’Ivoire *T. brucei* population. Clustering calculator (http://www.biology.ualberta.ca/jbrzusto/cluster.php) was used to create a Phylip Drawtree string (analysed using neighbor-joining clustering and Canberra Distances). A bootstrap with 100 iterations was also generated. This Drawtree string was then converted into a dendrogram using Figtree software (http://tree.bio.ed.ac.uk/). Heterozygosity, Nei’s genetic distance and F_ST_ were calculated using the GenAlEx software package for Microsoft Excel [41]. Hardy-Weinberg calculations were also performed using GenAlEx and linkage disequilibrium calculations were performed using GenePop v4.0.10 [45]–[47] and LIAN v3.5 [47] software packages. The likelihood of replicated genotypes occurring due to sexual recombination was examined using MLGsim 2.0 [48], using*P*_sex_ values calculated for each replicated MLG compared to 10,000 simulations for a population of the same size. Additionally, a principal component analysis (PCA) of the genetic distance was performed on the three populations using the GenAlEx software package [41]. *k*-means clustering was calculated from the eigenvalues from the PCA using the kmean() function of the R software package [49]. Finally, a sub-structuring modeling analysis was performed on the MLG using the STRUCTURE software package [50]. The models were formulated assuming an admixture model and correlated allele frequencies and a burn-in value of 50,000 and replication value of 250,000 [27], [28]. Several estimates of population size (K) were investigated for K = 2 to 12. The most likely estimate for K was inferred using both the posterior probability of 5 repeat runs for each value of K [40], [51], [52] and an estimate of delta K [40].

## Results

All studies of the population structure of group 1 *T. b. gambiense* to date have revealed foci to be largely clonal and relatively homogeneous [40]. Isoenzymes and RFLP analyses suggest that group 1 *T. b. gambiense* is genetically distinct from sympatric *T. b. brucei* and from group 2 *T. b. gambiense*. Here we have examined parasites isolated from both human patients and animals in four neighboring foci of infection in the Côte d’Ivoire in the late 1978 and 1983. The samples can be segregated into three different classes based on previous isoenzyme and RFLP studies and by the ability to resist lysis by human serum measured using the BIIT. Isolates that are invariably resistant to lysis and possess the classical isoenzymes of *T. b. gambiense* are classified as group 1 *T. b. gambiense*. A second group of parasites that also show resistance (either by BIIT or due to being isolated from a human host) but lack the isoenzyme markers characteristic of group 1 *T. b. gambiense*, are defined as non-gambiense or group 2 *T. b. gambiense*. Isoenzyme profiles of these isolates were highly variable [11]. The third group of isolates were sensitive to human serum in the BIIT and were not isolated from human patients; they were therefore classified as *T. b. brucei* and the isoenzyme profiles of these isolates were also highly variable [11].

### TgSGP Genotyping

**Figure 1:**
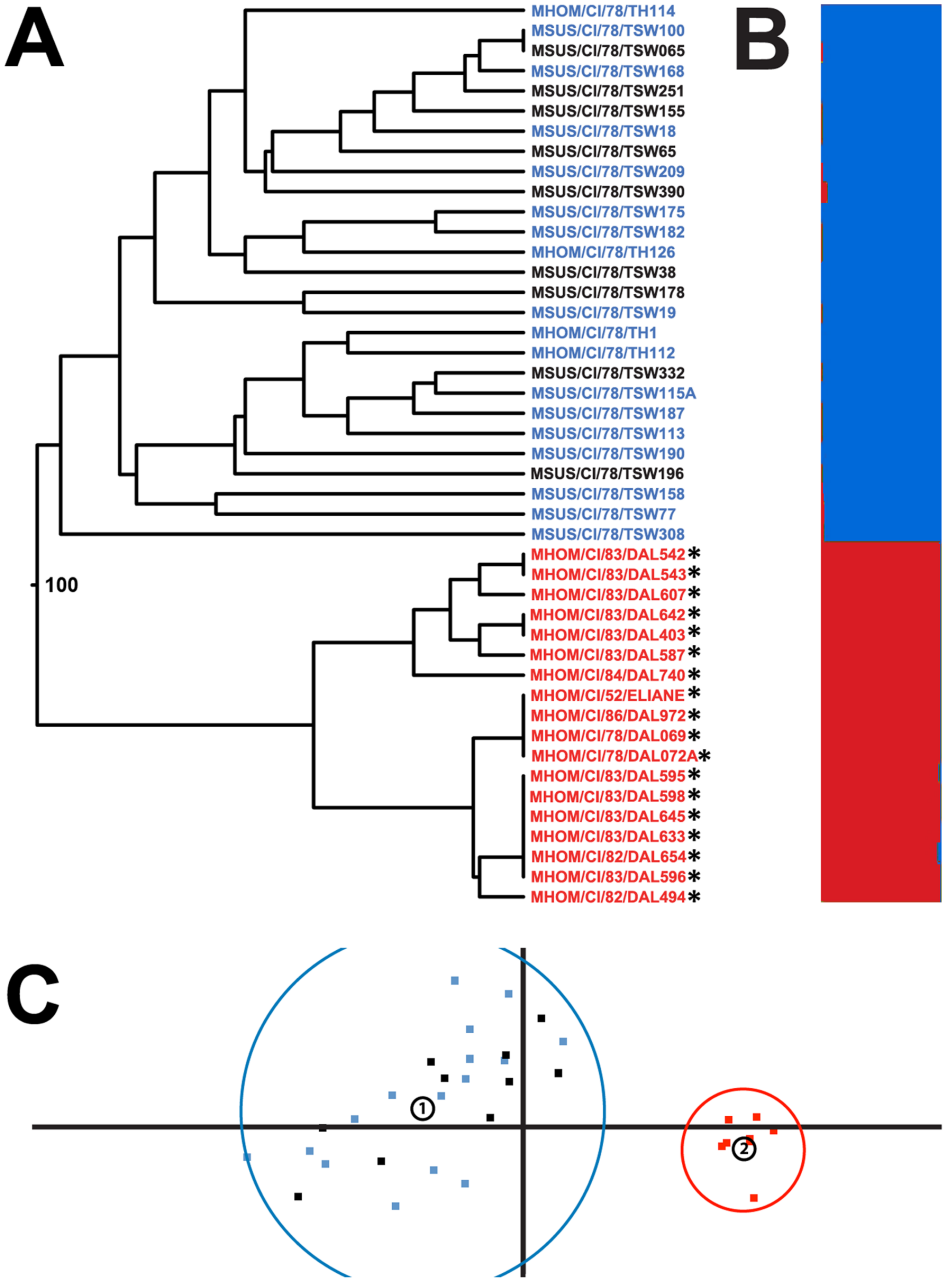
Genetic analysis of the *Trypanosoma brucei* population at the Côte d’ivoire focus. **a.** Dendrogram of multi-locus genotypes (MLG) for the *T. brucei* isolates collected from several townships in Côte d’Ivoire, over the period of time 1978–1983 in addition to DAL972 and ELIANE. Bootstrap values from 100 iterations are indicated for branch nodes with a bootstrap value above 10. The presence of *TgSGP* using primers spanning the 5′ and 3′ ends is indicated by *. Isolates that displayed human serum resistance, the classical *T. b. gambiense* isoenzyme profile and possess *TgSGP* can be inferred to be group 1 *T. b. gambiense* (Red). Isolates that display a degree of resistance or were isolated from humans but did not possess the classical isoenzyme profile or *TgSGP* were determined to be group 2 *T. b gambiense* (Blue). Strains that exhibited no human serum resistance and were isolated from animals are most likely *T. b. brucei* (Black). **b.** Predicted structure of the Côte d'Ivoire *T. brucei* focus for the most likely population number (K = 2). The proportion of each population that an isolate is a member of is indicated by red and blue in the histogram. **c.** Principal component analysis (PCA) of the Côte d’Ivoire *T. brucei* isolates using a pair-wise genetic distance comparison between each isolates MLG. The x-axis explains 56.45% of the variability in the populations and the y-axis 13.98%, for a total of 70.45%. Isolates are colored as outlined in 1a. The circled numbers indicate the centroids of the two clusters identified by *k*-means analysis. The limits of these clusters are also indicated.

The cell surface receptor TgSGP has been proposed as a diagnostic marker of group 1 *T. b. gambiense* [52] and PCR targeting the 3′ end of the gene was recommended to test for the presence of the gene as a diagnostic for *T. b. gambiense* group 1 [52]. Unfortunately, several group 2 *T. b. gambiense* are also positive for the published PCR diagnostic test, limiting its use in foci with both groups of *T. b. gambiense* [40]. Further work has now shown that the 3′ end of the *TgSGP* gene is closely related to the *VSG Tb10.v4.0178* and this *VSG* is likely the progenitor of this section of the *TgSGP* gene [47]. *Tb10.v4.0178* is present in several strains, including *T. b brucei*, *T. b. rhodesiense* and group 2 *T. b. gambiense* isolates [27], [28], so there is a possibility of false positives when using the originally proposed primers. In our analysis using the original primers targeted to the 3′ end of the TgSGP gene [27], [28], 21 isolates were positive for the *TgSGP* gene diagnostic, of which, 18 were previously characterized as group 1 *T. b. gambiense* on the basis of isoenzymes and RFLPs. However, two isolates (MSUS/CI/78/TSW209 and MSUS/CI/78/TSW158) that would be putatively identified as group 2 *T. b. gambiense* on the basis of human serum resistance phenotype in the BIIT but non-classical isoenzyme profiles, also showed positive for the 3′ end of the *TgSGP* gene. Additionally, a serum sensitive isolate (MSUS/CI/78/TSW308) was also positive. However, when using nested PCR primers that span both the 5′ and 3′ regions of the gene, the *TgSGP* gene was only shown to be present in the 18 isolates previously identified as group 1 *T. b. gambiense* (Figure 1a). This would suggest that the three non-group 1 *T. b. gambiense* isolates are false positives and possess either *VSG Tb10.v4.0178* or a closely related *VSG*. To investigate the relationships between the isolates from this disease focus, the isolates were further characterized using microsatellite genotyping.

### Microsatellite Genotyping

To investigate the relationships between the group 1 and 2 *T. b. gambiense* and *T. b. brucei* populations, the genotype of each of the 45 samples was determined using seven microsatellite markers. All seven markers are polymorphic, featuring several alleles at each locus (Table S1). For the entire sampled population, the majority of markers display a level of heterozygosity that is in in agreement with Hardy-Weinberg distribution (Table 2). Using these multi-locus genotypes (MLG), a dendrogram of relatedness was created to visualize the relationship between isolates in the study (figure 1a). The dendrogram revealed a discrete population containing only the 18 isolates characterized as group 1 *T. b. gambiense* by isoenzymes, RFLPs, BIIT and the presence of *TgSGP*. This clade also included the genome reference strain MHOM/CI/86/DAL972 and the well characterized group 1 strain MHOM/CI/52/ELIANE. The bootstrap value (100) indicates a high degree of support for the node that separates this group. The remaining 27 isolates formed a separate cluster distinct from the group 1 *T. b. gambiense,* containing both serum sensitive *T. b. brucei* (n = 9) and serum resistant group 2 *T. b. gambiense* isolates (n = 18). There is little bootstrap support for any of the branching nodes within this clade, suggesting this cluster represents a single population and that *T. b. brucei* and group 2 *T. b. gambiense* are largely indistinguishable from each other.

**Table 2:** Polymorphisms and heterozygosity of *T. brucei* at the Côte d’Ivoire focus (N = 45).

Locus	Alleles	ObservedHeterozygosity	Expected Heterozygosity
**Ch5/JS2**	8	0.667	0.784
**Ch11/110**	3	0.200	0.182
**Ch11/51**	3	0.356	0.299
**Ch1/18**	8	0.867	0.656
**Ch1/D2/7**	8	0.267	0.709
**Ch2/PLC**	5	0.200	0.424
**Ch3/IJ15/1**	6	0.359	0.363

Principal components analysis (PCA) was performed using the genetic distance of the isolates to create a multi-dimensional comparison (figure 1c). PCA identifies the independent factors from a data set that explain the maximum amount of correlation. Plotting these factors allows the structure of a population to be visualized. Further structure could be inferred by utilizing *k*-mean clustering and the eigenvalues from the PCA. This identified two distinct clusters; one containing *T. b. brucei* and group 2 *T. b. gambiense* populations and a second containing only isolates previously identified as group 1 *T. b. gambiense*. When analyzed by village, there is no evidence for any sub-structuring in the group 2 and *T. b. brucei* populations based on geography (data not shown) suggesting that the parasites within the townships are part of the same transmission cycle. Also, while not included in the analysis due to low sample numbers, similar genotypes are present in group 2 *T. b. gambiense* and *T. b. brucei* isolates from neighboring Burkina Faso, suggesting that there is one meta-population spanning the two countries (Table S1).

In order to further examine the population structure of the Côte d’Ivoire focus, STRUCTURE modeling was performed using the MLG data set. The most likely population number (K) was found to be 2 when estimated using both posterior probabilities and delta K (figure 1b) [47]. Isolates are either predominantly a member of a population containing only group 1 *T. b. gambiense* or a population containing group 2 *T. b. gambiense* and *T. b. brucei.* The removal of the group 1 isolates and then re-modeling with STRUCTURE did not indicate any further structure in the *T. b. brucei*/group 2 *T. b. gambiense* isolates (up to K = 12, data not shown).

### Population Analysis

In order to determine the population structure within each clearly distinct cluster as defined by the dendrogram, PCA and STRUCTURE, each cluster was examined separately. Cluster 1 contained the 18 isolates that had previously been identified as group 1 *T. b. gambiense* isolates and have subsequently been shown to all possess *TgSGP*. Cluster 2 contained all other isolates (n = 27), i.e. serum sensitive *T. b. brucei* and the non-classical gambiense isolates that showed any degree of serum resistance and could be termed group 2 *T. b. gambiense*. To further examine the differences between the sub-species and groups, cluster 2 was sub-divided into those isolates that previously showed any degree of resistance in a BIIT (putative group 2 *T. b. gambiense*, n = 18) and those that were highly sensitive and could be described as *T. b. brucei* isolates (n = 9).

The relationship between these populations was analyzed using two statistical tests, Nei’s genetic distance and F_ST._ Both Nei’s genetic distance and the F_ST_ statistic indicate that both populations within cluster 2 are distinct from cluster 1 and that within cluster 2, there is very little genetic difference between *T. b. brucei* and group 2 *T. b. gambiense* populations (Tables 3 & 4). As there is so little difference between populations, subsequent analysis considered the cluster 2 as a single population.

**Table 3:** Nei’s genetic distance between the populations of group 1 and 2 *T. b. gambiense* and *T. b. brucei* at the Côte d’Ivoire focus.

	*T. b. brucei* (N = 9)	Gp 2 *T. b. gambiense* (N = 16)	Gp 1 *T. b. gambiense* (N = 20)
***T. b. brucei***	0.000	–	–
**Gp 2 ** ***T. b. gambiense***	0.026	0.000	–
**Gp 1 ** ***T. b. gambiense***	0.357	0.321	0.000

**Table 4:** F_st_ proportion indicating genetic distance between the populations of group 1 and 2 *T. b. gambiense* and *T. b. brucei* at the Côte d’Ivoire focus.

	*T. b. brucei* (N = 9)	Gp 2 *T. b. gambiense* (N = 16)	Gp 1 *T. b. gambiense* (N = 20)
***T. b. brucei***	0.000	–	–
**Gp 2 ** ***T. b. gambiense***	0.138	0.000	–
**Gp 1 ** ***T. b. gambiense***	0.277	0.264	0.000

If random mating is occurring within the Côte d’Ivoire population then the number of heterozygous genotypes within the population should be a predictable proportion based on the number and frequency of alleles present in the population, in agreement with Hardy-Weinberg expectations. Analysis of the genotype frequencies in cluster 2 (the combined *T. b. brucei* and group 2 *T. b. gambiense* isolates) revealed that none of the seven markers significantly deviated from Hardy-Weinberg equilibrium (Table 5). These data are consistent with the hypothesis that some degree of mating is occurring within this cluster i.e. within and between the group 2 *T. b. gambiense* and *T. b. brucei*. In contrast, analysis of markers for cluster 1 shows that the group 1 *T. b. gambiense* population display a strong deviation from Hardy-Weinberg, with markers being either monomorphic or possessing heterozygote excess strongly disagreeing with the Hardy-Weinberg hypothesis (Table 6).

**Table 5:** Hardy-Weinberg analysis for the combined population of group 2 *T. b. gambiense* and *T. b. brucei* at the Côte d’Ivoire focus (N = 25).

Locus	DF	?^2^	Probability	Significance
Ch5/JS2	15	21.021	0.136	Not Significant
Ch11/110	3	1.080	0.782	Not Significant
Ch11/51	3	0.173	0.982	Not Significant
Ch1/18	15	18.798	0.223	Not Significant
Ch1/D2/7	6	6.762	0.343	Not Significant
Ch2/PLC	10	15.693	0.109	Not Significant
Ch3/IJ15/1	6	6.832	0.559	Not Significant

**Table 6:** Hardy-Weinberg analysis of group 1 *T. b. gambiense* isolates at the Côte d’Ivoire focus (N = 20).

Locus	DF	?^2^	Probability	Significance
Ch5/JS2	3	18.000	0.000	P<0.001
Ch11/110	Monomorphic	–	–	–
Ch11/51	1	4.500	0.034	P<0.05
Ch1/18	1	18.000	0.000	P<0.001
Ch1/D2/7	6	18.147	0.006	P<0.001
Ch2/PLC	1	0.015	0.904	Not Significant
Ch3/IJ15/1	Monomorphic	–	–	–

A second method to assess the degree of mating within cluster 2 is to estimate the amount of linkage disequilibrium in allele and genotype frequencies. The presence of mating in a population will cause alleles at unlinked loci to be inherited in a randomly assorted manner, leading to linkage equilibrium, while a population with little mating will exhibit linkage disequilibrium. Analysis of linkage equilibrium revealed that the overwhelming majority of allele combinations do not show significant linkage disequilibrium in cluster 2 (Table 7). Additionally, both the *T. b. brucei* and group 2 *T. b. gambiense* populations exhibit a standardized Index of Association (I_A_) that tends towards zero. I_A_ s a function of the rate of recombination and is zero for complete linkage equilibrium, so our data are consistent with linkage equilibrium existing in these populations (Table 8). Linkage equilibrium is also exhibited when the *T. b. brucei* and group 2 populations are analyzed together, suggesting linkage equilibrium between the populations. These data, combined with the Hardy-Weinberg analysis suggest that there is a degree of mating and re-assortment both between and within the *T. b. brucei* and group 2 *T. b. gambiense* populations. The group 1 *T. b. gambiense* alleles are largely monomorphic or uninformative so a linkage disequilibrium study is not possible with this population. However the low number of alleles and limited variability correlates with studies of other foci, suggesting that group 1 *T. b. gambiense* possesses a clonal population structure that is separate from that of the group 2 *T. b. gambiense* and *T. b. brucei* population [27], [28]. The low variability in genotypes of the group 1 *T. b. gambiense* isolates were tested for clonality using MLGsim. This indicated that all observed *P*_sex_ values for each MLG expansion were highly significant at the *p*<0.01 threshold, suggesting that all of the samples are clonal. Conversely, the same analysis on the *T. b. brucei* and group 2 *T. b. gambiense* populations shows that most genotypes have likely arisen due to sexual recombination and only two of the samples possess possible clonal genotypes at the *p*<0.01 significance threshold, MSUS/CI/78/TSW158 and MSUS/CI/78/TSW175.

**Table 7:** Linkage disequilibrium for each genetic marker (statistically significant disequilibrium highlighted in bold) between pair wise polymorphic loci in the combined *T. b. brucei* and group 2 *T. b. gambiense* population at the Côte d’Ivoire focus (N = 25).

Locus 1	Locus 2	Probability	Standard Error
Ch5/JS2	Ch11/110	0.345	0.014
Ch5/JS2	Ch11/51	0.053	0.006
Ch11/110	Ch11/51	1.000	0.000
Ch5/JS2	Ch1/18	0.299	0.029
Ch11/110	Ch1/18	0.136	0.008
Ch11/51	Ch1/18	0.410	0.012
Ch5/JS2	Ch1/D2/7	0.172	0.019
Ch11/110	Ch1/D2/7	0.351	0.009
Ch11/51	Ch1/D2/7	1.000	0.000
**Ch1/18**	**Ch1/D2/7**	**0.040**	**0.008**
Ch5/JS2	Ch2/PLC	0.081	0.012
Ch11/110	Ch2/PLC	0.648	0.003
Ch11/51	Ch2/PLC	0.573	0.018
**Ch1/18**	**Ch2/PLC**	**0.007**	**0.032**
Ch1/D2/7	Ch2/PLC	0.293	0.010
Ch5/JS2	Ch3/IJ15/1	0.373	0.015
Ch11/110	Ch3/IJ15/1	0.237	0.022
Ch11/51	Ch3/IJ15/1	0.363	0.021
Ch1/18	Ch3/IJ15/1	0.721	0.023
Ch1/D2/7	Ch3/IJ15/1	0.274	0.011
Ch2/PLC	Ch3/IJ15/1	0.525	0.021

The data were analyzed using the Genepop 4.0 software package.

**Table 8:** Standardised indices of association (I_A_) for the populations of group 1 and 2 *T. b. gambiense* and *T. b. brucei* at the Côte d’Ivoire focus.

Population analyzed	V_D_-	V_E_-	I_A_
**Total Population (N = 45)**	2.739	1.476	0.143
**Combined ** ***T. b. brucei*** ** & gp 2** ***T. b. gambiense*** ** (N = 25)**	1.686	1.311	0.048
**Gp 2 ** ***T. b. gambiense*** ** (N = 16)**	1.716	1.227	0.067
***T. b. brucei*** ** (N = 9)**	1.441	1.360	0.099
**Gp 1 ** ***T. b. gambiense*** ** (N = 20)**	–	–	∞

Data were analyzed using the LIAN software package to test the null hypothesis of linkage equilibrium. This null hypothesis is that the variance of loci that do not show linkage (V_D_^–^) is equal to the expected variance of loci modelled under linkage equilibrium (V_E_^–^).

It would appear from these data that the *T. b. brucei* and group 2 *T. b. gambiense* populations exhibit some degree of mating, both within and between sub-species. The group 1 *T. b. gambiense* population is clonal and distinct from the sympatric *T. b. brucei* and group 2 *T. b gambiense* populations.

## Discussion

These data reinforce studies from other foci that populations of group 1 *T. b. gambiense* are likely clonal [27], [28]. The reasons for the clonality of group 1 *T. b. gambiense* are unclear as the genes necessary for meiosis are present [37] and are expressed during tsetse infection (Peacock and Gibson, unpublished data). Another possible reason for the low frequency of mating observed in group 1 *T. b. gambiense* is that, due to the limited number of genotypes present, the chances of a mixed genotype infection occurring in the tsetse vector is unlikely. This precludes any mating other than selfing, which has been observed in laboratory crosses for *T. b. brucei* [53]. However a high frequency of selfing would result in a population with an excess of homozygotes rather than the observed excess of heterozygotes, and is therefore unlikely.

The observed homogeneous population of group 1 *T. b. gambiense* could also be due to a bottleneck effect that occurred in the past or if group 1 *T. b. gambiense* underwent clonal expansion, possibly after it gained the human infectivity trait. Despite questions as to how the clonal population structure has developed, these data show that the group 1 *T. b. gambiense* genome reference strain MHOM/CI/86/DAL972 is clearly a good representative of group 1 strains from this area. Previous studies have shown that the MLG in the populations of various group 1 *T. b. gambiense* foci are significantly different from each other [27] and examination of the microsatellite alleles in this study with previous studies indicates that there are few shared alleles at each marker. Indeed, the necessity to include new markers specific to this study reinforces the fact that the focus possesses a different clonal population of group 1 *T. b. gambiense* to that found in other foci.

This study has also shown that the STIB386 strain (MHOM/CI/78/TH114) used in genetic mapping studies [54] is a good representative for Côte d’Ivoire group 2 *T. b. gambiense* in that it is distinct from the group 1 *T. b. gambiense* population and clusters with other group 2 *T. b. gambiense* strains. Previous studies have suggested that group 2 *T. b. gambiense* are more varied than group 1 and more similar to *T. b. brucei* [16], [19], [22]–[26]. The data presented here show that not only was there a large and genetically varied breeding population of group 2 *T. b. gambiense* at the Côte d’Ivoire focus, that these individuals were almost certainly breeding with local *T. b. brucei*. The *T. b. brucei* and group 2 *T. b. gambiense* populations are very similar, suggesting that they form one breeding population. There is a suggestion that this breeding population extends to neighboring Burkina Faso, as similar microsatellite alleles are found in both countries. However, this may be due to the seasonal, economic migration of the Mossi people between the two nations [55] that transports parasites rather then a large contiguous population. Nevertheless, group 2 *T. b. gambiense* can be seen as an extended host range variant *T. b. brucei*.

The degree to which mating occurs in a population is an important consideration due to the process acting as a major source of variation. While the human infectivity trait present in group 2 *T. b. gambiense* may have evolved once, it is now present in a wide range of genetic backgrounds. This is in contrast to group 1 *T. b. gambiense* which has limited genetic diversity at each discrete disease focus and hence a lower capacity to evolve. The genetic backgrounds of the group 2 *T. b. gambiense* infectivity trait may be in constant flux due to evidence suggesting the presence of mating within the population. This may go someway to explaining the emergence of a “new” group of *T. b. gambiense* in Côte d’Ivoire [56]. This new human infective group has been claimed as distinct from both group 1 *T. b. gambiense* and group 2 *T. b. gambiense* from the early 1980s. However, based on the heterogeneity and propensity for mating displayed by group 2 in our study, it is likely that this new group belongs to group 2, with the group 2 human serum resistance mechanism on a new genetic background. Unfortunately, the human serum resistance profile of the new group has not yet been described and it is unknown whether it also displays a variable, non-*SRA* mediated resistance [56]. Elucidation of the resistance mechanism for group 2 *T. b. gambiense* would also allow this hypothesis to be investigated.

A previous microsatellite study has indicated that although group 2 *T. b. gambiense* from Côte d’Ivoire is related to *T. b. brucei*, it also clusters close to group 1 *T. b. gambiense* [29]. This has led to the suggestion it is a hybrid of *T. b. brucei* and group *1 T. b. gambiense*. However, the *T. b. brucei* used in the study were mostly from East Africa and are not be representative of *T. b. brucei* in Côte d’Ivoire. Our data clearly shows that the West African *T. b. brucei* and group 2 *T. b. gambiense* populations are largely indistinguishable and that group 2 is not closer to group 1 *T. b. gambiense* than local *T. b. brucei*. There is no evidence to support the hypothesis that group 2 *T. b. gambiense* is a hybrid of group 1 *T. b. gambiense* and *T. b. brucei* and that this sub-species has evolved separately. We have also shown that *TgSGP* is not present in the local group 2 *T. b. gambiense* or *T. b. brucei* populations by using nested primers that span the 5′ and 3′ ends of the gene. While this is counter to other published data [40], the primers described in the original diagnostic PCR are likely to show false positives due to similarity to the 3′ end of *TgSGP* and the *VSG Tb10.v4.0178* [52]. This observation adds further weight to the hypothesis that there is no mating between group 1 or group 2 *T. b. gambiense*, and that group 2 has not emerged as a result of hybridizing event.

The study presented here, in conjunction with other published data, suggests that human infectivity has arisen on at least three occasions (with some evidence of a fourth variant). In East Africa the evolution of *SRA* and the high levels of mating occurring there have allowed the human infectivity trait to spread through the *T. brucei* population, generating the *T. b. rhodesiense* sub-species [57], [58]. However, there are also human infective trypanosomes in this area that do not possess *SRA,* suggesting a second novel mechanism [3], [59]. In West and Central Africa, group 1 *T. b. gambiense* is the dominant cause of human African sleeping sickness. This group of parasites has evolved a constitutively expressed human serum resistance mechanism that does not depend on *SRA.* After evolution of this trait, the sub-species appears to have expanded clonally in the human population. Just as in East Africa, there is also a human infective *T. brucei* population separate from the dominant form that appears to have a novel resistance mechanism. This group 2 *T. b. gambiense* population appears to be a host range variant of *T. b. brucei* and the human infectivity trait it possesses is freely transmitted among the local *T. b. brucei* population. The presence of mating amongst group 2 *T. b. gambiense* and *T. b. brucei* strains makes evolution of new traits more likely and may allow the human infectivity trait to be combined with new phenotypes that may increase its virulence. It also allows the human resistance trait to persist in the local *T. brucei* animal reservoir through genetic transfer. Understanding whether the inter-sub-species mating and transfer of human infectivity traits exhibited in Côte d’Ivoire can also occur in other foci is becoming more pressing. In Uganda, the *T. b. gambiense* and *T. b. rhodesiense* foci are moving closer together and their ranges will begin to overlap [60], [61]. Fortunately the possibility of mating between group 1 *T. b. gambiense* and *T. b. rhodesiense* appears unlikely due to the clonal nature of group 1 *T. b gambiense* [27], [28], and while *T. b. rhodesiense* mating with group 2 *T. b. gambiense* is certainly possible [33], the ranges of these two diseases are not converging at present. Taken together, all of these data indicate that *T. brucei* has a high zoonotic potential despite specific trypanolytic countermeasures that have been inherited by humans.

## Supporting Information

Table S1 **Overview of sample origin, host, human serum resistance phenotype and alleles present for each microsatellite marker.** The presence absence of *TgSGP* is also indicated, whether it possesses the full gene (1) or the progenitor VSG (2).
(XLSX)
